# Supplementary material for: Using Cholesterol-Loaded Cyclodextrin to Improve Cryo-Survivability and Reduce Capacitation-Like Changes in Gender-Ablated Jersey Semen
Source: Animals (Basel). 2025 Jul 11;15(14):2038. doi: 10.3390/ani15142038 (PMC12291860; doi:10.3390/ani15142038)
Supplement: Supplementary file 1 [file animals-15-02038-s001.zip › animals-3681081-supplementary.pdf]

Supplementary Table S1. Fluorescent dyes: emissions, excitations, and detection filters used in the current study.

Dye	Why	Peak Excitation Wavelength (nm)	Peak Emission Wavelength (nm)	Excitation Laser Wavelength (nm)	Excitation Laser Power (mW)	Emission Band Pass Filter (nm)	Emission Long Pass Dichroic Mirror (nm)
Sex selection Hoechst	Gate out non-sperm events	350-352	454- 461	355	20	450/50	410
PI	Gate out dead cells or evaluate % dead	535	615-617	561	100	610/20	600
FITC-PNA	Acrosome integrity	495-500	514-521	488	20	530/30	505
Fluo-4 AM	Calcium level	480-494	506-525	488	20	530/30	505
JC-1 monomers (green)	Low mitochondrial potential	514	529	488	20	530/30	505
JC-1 aggregates (orange)	High mitochondrial potential	485-585	590	488	20	582/15	550
M540	Membrane stability and fluidity	561	579	561	100	610/20	600
YoPro-1	% dead	491	509	488	20	530/30	505
